# Confinement, Comfort and Health: Analysis of the Real Influence of Lockdown on University Students during the COVID-19 Pandemic

**DOI:** 10.3390/ijerph18115572

**Published:** 2021-05-23

**Authors:** Antonio Millán-Jiménez, Rafael Herrera-Limones, Álvaro López-Escamilla, Emma López-Rubio, Miguel Torres-García

**Affiliations:** 1 Faculty of Medicine, University of Seville, Avda. Sánchez Pizjuán, s/n, 41009 Seville, Spain; amillan1@us.es (A.M.-J.); emma.lopez.rubio@gmail.es (E.L.-R.); 2 Institute of Architecture and Building Sciences, Superior Technical School of Architecture, University of Seville, Av. Reina Mercedes 2, 41012 Seville, Spain; alvlopesc@alum.us.es; 3 Energy Engineering Department, Superior Technical School of Engineering, University of Seville, Camino de los Descubrimientos s/n, 41092 Seville, Spain; migueltorres@us.es

**Keywords:** COVID-19, health, comfort, university students, housing, survey

## Abstract

The COVID-19 pandemic forced the population worldwide into lockdown. The purpose of this study was to assess the impact of this measure on the health and comfort of university students and the role that the characteristics of the home may have played. It is essential to differentiate between the terms comfort and health both from the medical and architectural perspectives, as there are differences between the two concepts that are, nonetheless, shared by both disciplines. An online survey was fulfilled by 188 medicine and architecture undergraduate students at the University of Seville, Spain. In terms of health, 89% suffered neuropsychiatric disorders (56% anxiety and 49% depression), 38% gained weight and 59% reported alcohol consumption. In relation to comfort, the majority rated their home positively, comfortable in terms of room temperature and noise at night, and they had a good relationship with cohabitants. However, those who did not have a balcony or terrace would have liked to have open spaces They would have also liked to increase the size of their bedroom, where they spent most of their time and where they studied. A built-up environment gave them a sense of being imprisoned, while those who enjoyed open spaces found a sense of peace. The absence of open spaces in the house, the environment and the impossibility of making the most frequently used spaces more flexible may have had negative impacts on the health and comfort of university students during confinement.

## 1. Introduction

The COVID-19 pandemic led health authorities in the majority of countries around the world to adopt a series of measures to combat and control the spread of the virus. Among these, perhaps the most extreme yet efficient [1,2] was a complete lockdown over a number of weeks, as was the case in Spain.

Home confinement, one of the principal measures taken in the fight against the COVID-19 coronavirus pandemic, led to a substantial change in the use of homes. People found themselves sharing a space 24 h a day with family members who they would normally only come into contact with for sleeping, eating, and sharing some moments in the evening.

This new experience has most likely had an impact on both the physical and mental health [3], as well as the comfort, of confined undergraduates [4,5] as a consequence not only of the severity of the measures, but also of a lack of exercise [6] and privacy, changes in study and work environments, changes in eating habits [7], and a lack of natural light in homes without balconies and private gardens. These changes in lifestyles [8] can be detrimental to an individual’s health [9,10].

In Spain, this exceptional situation made it an obligation by law to stay 24 h a day, 7 days a week inside a dwelling not designed for that purpose. People usually use their homes intermittently throughout a 24 h period: for sleeping at night, mealtimes, and other part-time activities such as some professional activities, studying, reading, and leisure activities associated with listening to music, and watching TV.

This pandemic is certain to transform many aspects of our lives [11,12,13], including homes and, as a consequence, cities [14], just as occurred in previous pandemics in history [15]. The increased use of homes has meant that inhabitants have experienced their deficiencies [16] and, as a consequence, have become more critical with their space [17].

Nevertheless, these conditions, which, from an ethical standpoint, would never have been included in the design of a science experiment, offers a unique opportunity to analyse the conditions of comfort and health among people confined in their homes. They also lead to a better understanding of how the design of homes and the habitats in which they are built affect the two aspects analysed: comfort and health of inhabitants. The scientific community specialised in studying dwellings now has the opportunity and the obligation to rethink the relation between medicine and architecture, and more specifically, between the day-to-day human habitat and health [18]. It is therefore appropriate to reappraise the aspects related to interior comfort in homes both for normal use and the more specific use that has arisen as a result of the lockdown [19].

As this article will go on to show, the impact that architecture has on the health of inhabitants is not exclusively related to quantifiable parameters used to evaluate a space such as temperature, humidity, lighting or air quality [20,21,22,23,24,25]. Numerous studies have confirmed that the comfort of an inhabitant is also strongly related to materiality, decoration and the views from windows, for example [26,27,28,29,30,31]. A recent study, carried out with university students in Valladolid (Valladolid Spain), demonstrated the negative impact that confinement has had on the mental health of students and teachers. However, the study did not analyse the characteristics of the house where they were confined and their possible influence on the results obtained [32,33].

The aim of this study was to discover whether the use of a home during confinement (that is to say, an intensive, almost 24 h-a-day use) has affected the health and comfort of final-year Medicine and Architecture undergraduates from the University of Seville (US), Spain. This is a sample of the population who, due to their age, the city’s climatic environment and their condition as Latinos, are accustomed to a way of life that is totally different from that imposed by confinement. Aspects related to housing and the individual were analysed in order to provide an answer to the question posed [34,35,36].

The consequences suffered by any other person in these extreme conditions are amplified for the particular cohort of this study, on the one hand, by the particularities of youth and, on the other, by the vision that these budding doctors and architects have of health and housing aspects, conditioned by the type of housing in which they have had to carry out their confinement (which has definitely shown design deficiencies). Special consideration was paid to aspects of health which may have been affected by a lack of exercise, reduction in exposure to sunlight, weight loss and gain, mood swings and changes to diet. Data were collected prior to and during confinement.

With regard to comfort, aspects such as sound proofing, night-time rest, leisure activities, privacy and time spent using social media and online games were taken into consideration.

The secondary objective was to evaluate housing architecture under conditions it was not designed for. This objective was studied by obtaining information regarding the type of housing, number of rooms, work or study space and the conditions in which these were carried out, open spaces (balcony, patio etc.), the distribution of space and quality of materials (floor and windows).

The study sample of final-year Medicine and Architecture undergraduates was considered of a homogeneous age and academic level and would, therefore, avoid bias in the interpretation of the results obtained. By comparing students from two different academic disciplines, a complementary vision of the analysed aspects would be achieved. Another factor for the choice of sample was its accessibility in the context of the University of Seville, making this project feasible.

## 2. Materials and Methods

### 2.1. Context of the Study

The methodology employed in this study was the result of an ongoing process undertaken by members of the University of Seville HUM-965 Research Group to find an optimal, reliable methodology for grading comfort and its impact on the health of individuals [37]. This methodology has been tested in various editions of the Solar Decathlon, the housing prototype competition in which the University of Seville has participated [38,39,40].

The Solar Decathlon is the most important international architecture and sustainable building competition [41,42], aimed at university students and funded by private businesses. This contest, based on learning by doing, is the ideal tool for the completion of university studies before embarking on a career [43,44,45,46].

**Figure 1:**
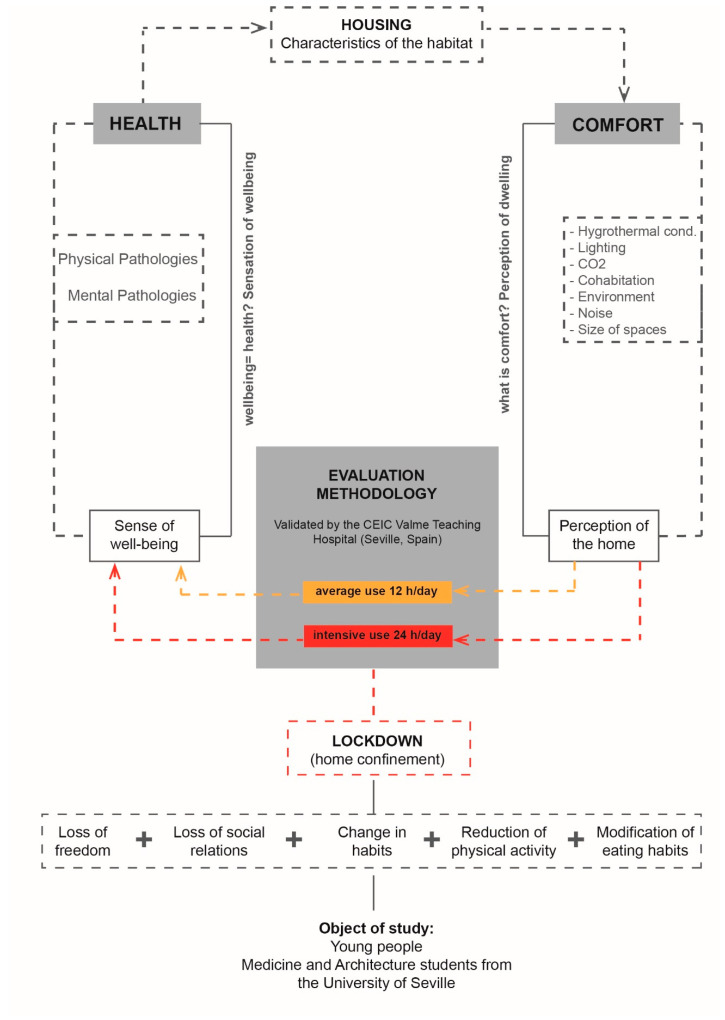
Diagram of the analysis carried out.

The contest allows universities and businesses to develop and test initiatives related to human dwelling spaces, from energy optimisation through the use of different high-efficiency systems and passive design [47,48,49,50,51,52], to the analysis of construction elements employed in every solution [53,54,55], as well as using different home automation tools or studying the comfort conditions of the dwelling [56], and, as a result, their effect on the health of the user (Figure 1).

### 2.2. Sample and Type of Research

This article presents an observational, descriptive and transversal study based on a survey [57,58] which does not analyse specific aspects such as the effect of weight on health, or noise on comfort in the home. It seeks to provide a global vision of a considerable quantity of analysable data to discover whether health and comfort have been affected generally, and more concretely, what aspects have been most affected by this possible alteration.

The survey consisted of 48 questions grouped under the headings of demographic data (date of birth, sex, and type of studies); health-related data (weight, height, personal history, weight gain or loss, consumption of toxic substances, illnesses or symptoms during lockdown); data related to comfort (number of trips to the street, noise, hours of natural light, leisure activities, exercise, thermal comfort, relationship with residents and neighbours, and sensation when looking out of the window) and directly related to the dwelling (type of dwelling, size, presence of open spaces, place of study, environment and modifications he/she would make to the dwelling).

The anonymous survey was carried out at the end of Spain’s lockdown period between the 30 June and 15 July. The sample invited to participate in the survey were final-year Medicine and Architecture undergraduates at the University of Seville. This group was made up of a total of 560 students: 350 medical students and 210 architecture students. Invitations to take part were sent via the “message students” tool on the University of Seville’s Blackboard platform. This communication channel allows information, in this case, a survey, to be sent to a determined group of students without using individual email addresses. No selection of recipients was made, neither was student identification possible from the results obtained via Google Forms^®^, as no personal information such as name, enrolment number, telephone number or email address was required. The only figure available was the number of answers received out of the total number of recipients.

The Google Forms^®^ tool was used to design the 48 multiple-choice, rating-scale, and open-ended question survey. To process the data, the IBM^®^ SPSSS^®^ Statistics version 25 program for Macintosh was used.

Quantitative variables were summarised with means and standard deviations and qualitative variables with percentages. Qualitative variables were analysed using the chi-square test and Fisher’s exact test. Confidence intervals at 95% were obtained to quantify the difference in percentages. Quantitative variables were compared using the Student’s *t*-test.

The characteristics of the dwelling used during the period of confinement would help provide the aspects which have had effects on comfort and health and, more widely, the habitat in which it is located. This analysis could uncover design deficits or potential areas for improvement in future constructions.

### 2.3. Ethical Aspects

Alongside the survey, all participants received detailed information about the study, its objectives and the confidentiality of the processed data. Participants were required to give their consent before completing the survey.

The study was approved by the Valme Teaching Hospital’s Clinical Research Ethics Committee, which was designated as an ethics committee of reference by the University of Seville on 30 June 2020.

## 3. Results

**Figure 2:**
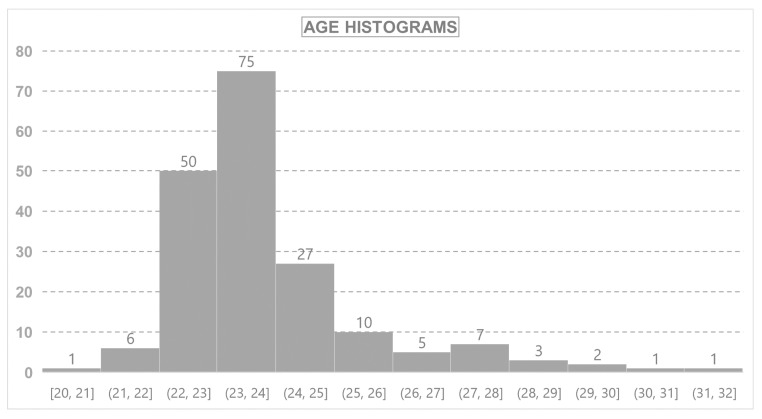
Age histograms.

From the 560 students enrolled in the final year of both subject areas, results were received from 188 (32%). The characteristics of participants are shown in Table 1 and Figure 2. No significant differences were observed in the responses according to gender or studies.

### 3.1. Results in Health

All participants were healthy at the time of the survey. The average age was 24.55 (DE 2.29) and 68% were female. The majority were studying medicine (70%). BMI values were normal in 72% of participants, overweight in 17.1% and obese in 2.1%.

In relation to weight loss and gain, 30% of those surveyed maintained the same weight, 38% gained weight, and 32% lost one or two kilograms.

On the other hand, 90.6% showed symptoms or fell ill during the lockdown, with the most prevalent being neuro-psychiatric pathologies (Table 2). However, only 11.7% required medical assistance. In addition, 62% had consumed alcohol, tobacco, hashish, marijuana or several of these.

### 3.2. Results in Comfort

Results in comfort were evaluated in the following sections:

#### 3.2.1. Soundproofing and Natural Light in the Home

Participants evaluated night-time and daytime noise on a 4-band numeric scale: from 0 to 1 (no noise); from 2 to 4 (low noise); from 5 to 6 (moderate); from 7 to 8 (substantial). Hours of daylight and the need for electric lighting are important in terms of comfort and health in the home (Table 3).

#### 3.2.2. Privacy and Social Relations

The evaluation of privacy was based on the characteristics of the bedroom as, if it is a shared space, this is where privacy can be compromised. The results showed that 86% of respondents had their own bedroom while 14% shared with another person.

Social relations complete the sensation of comfort as they offer the possibility to socialise and avoid personal isolation, which is a negative aspect or discomfort. The majority (84%) lived with their family and the average number of cohabitants was 3 or 4 people. With regard to social relations, 91% had a good or very good relationship with their cohabitants. Amongst neighbours, this percentage fell to 41%, with 37% recognising they had a bad relationship with them.

#### 3.2.3. Leisure Activities and Exercise

**Figure 3:**
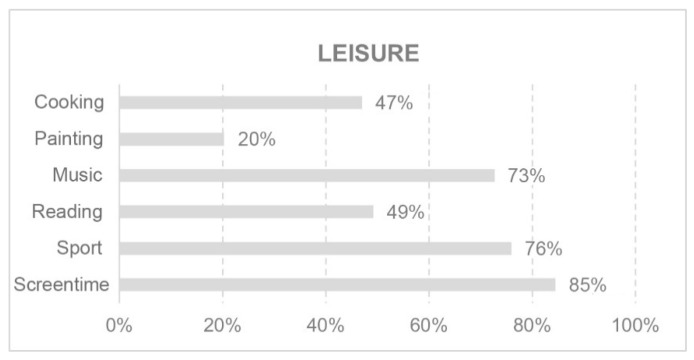
Leisure.

**Figure 4:**
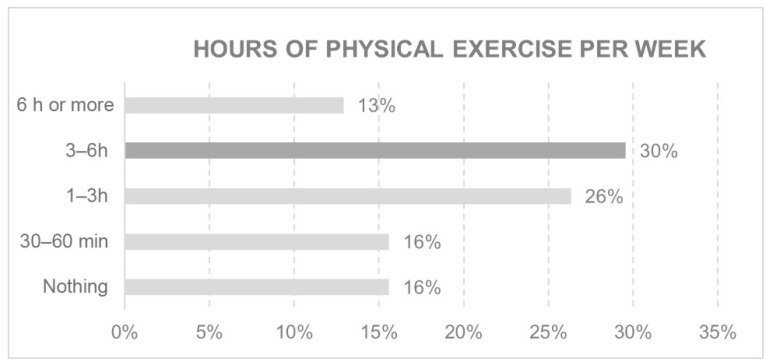
Hours of physical exercise per week.

In today’s society, leisure activities and exercise are considered part of an individual’s wellbeing or comfort. Time spent on these activities forms part of our routine. These aspects are shown in Figure 3 and Figure 4.

#### 3.2.4. Adequacy of Study Space

**Figure 5:**
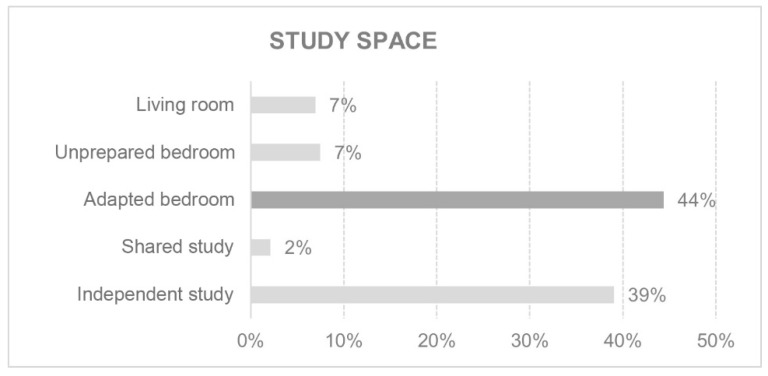
Study space.

**Figure 6:**
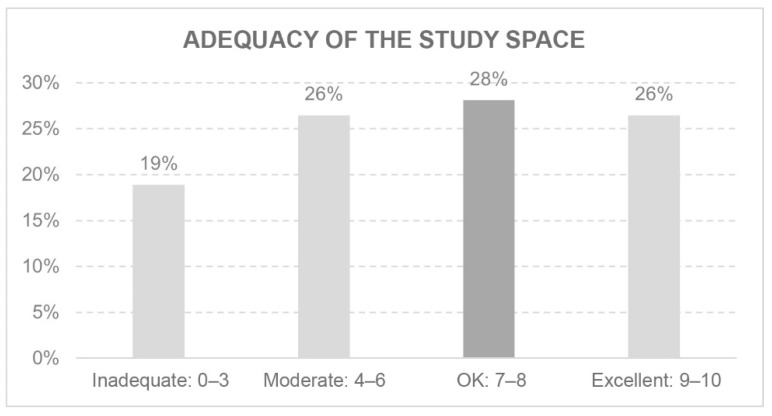
Adequacy of the study space.

Many hours a day are spent in the space designated for work or study. Whether or not this space is considered adequate influences the overall level of comfort (Figure 5 and Figure 6).

#### 3.2.5. Thermal Sensation during Confinement

One of the aspects that has possibly the greatest impact on the comfort of a dwelling is thermal sensation. For the majority of those surveyed (93%), this was comfortable, while 4% felt hot and 3% felt cold. The heating or air conditioning system affects this sensation. These are reported in the section which covers housing types.

### 3.3. Housing-Related Results

The results obtained regarding the evaluation of the dwelling during lockdown are recorded in the following sections.

#### 3.3.1. Overall Evaluation of the Home

**Figure 7:**
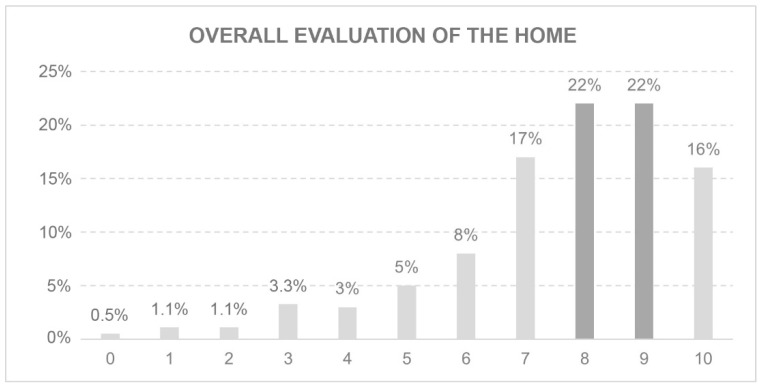
Overall evaluation of the home.

The response to this question aims to identify the overall level of satisfaction of the occupants of a dwelling during lockdown. A scale of 0 to 10 was used, with 0 being the least favourable and 10 the most satisfied. Notably, 77% of respondents gave an evaluation of 7 or more (Figure 7). Nevertheless, the evaluation of a single-family house was significantly higher (8.04) than that of a flat (6.7) (p 0.000; IC −1.93–−0.72).

#### 3.3.2. Type of Dwelling

In the responses to this question, 83% of those surveyed were confined in the family home. This could have had an influence on the type of dwelling as, under normal circumstances, students live in shared flats or halls of residence. In this study, 52% lived in single-family houses and 48% in flats.

#### 3.3.3. Characteristics of the Dwelling

Regarding the design of the houses, 50% had three bedrooms and two bathrooms. In terms of heating and cooling systems, 56% of the participants had air conditioning in some rooms, 32% central air conditioning and 36% electric radiators. Most of the dwellings (79.7%) had cross ventilation.

Some publications highlight the importance of good cross ventilation, especially during the winter months. There has been an increase in the concentration of unhealthy chemical contaminants in the home due to the need to save energy and the intense disinfection of homes [59].

#### 3.3.4. Environment

**Figure 8:**
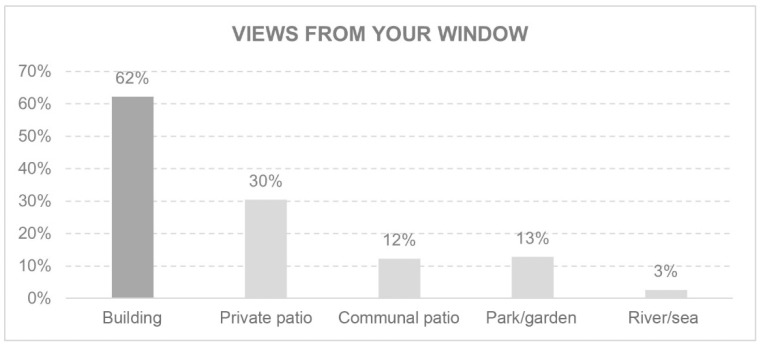
Views from your window.

**Figure 9:**
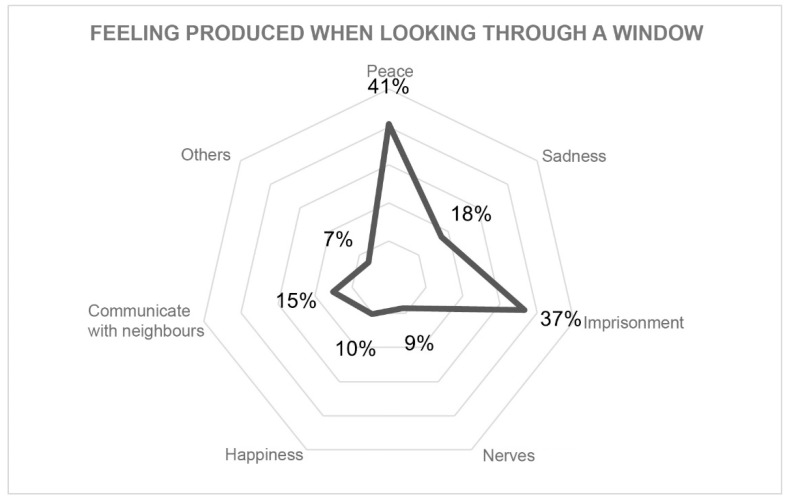
Feeling produced when looking through a window.

**Figure 10:**
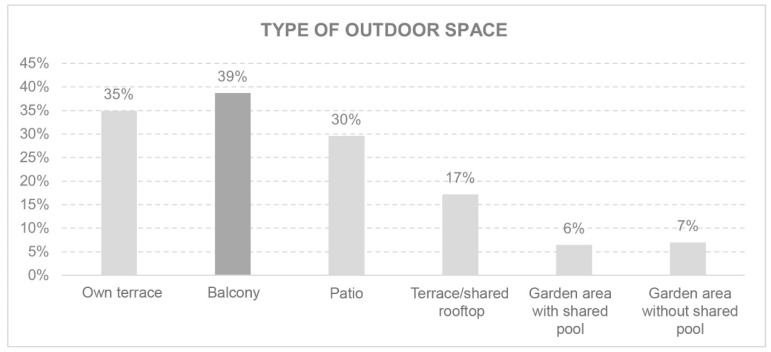
Type of outdoor space.

The environment was evaluated by the views from the windows (Figure 8) and the feelings these views produced in inhabitants (Figure 9). This section also considered the existence of open-air spaces and their characteristics (Figure 10).

The feeling of imprisonment is significantly linked to the views of other buildings from a window (p 0.005; IC 0.06–0.34). This feeling was not related to the type of dwelling or university studies. On the other hand, the feeling of peace when looking out of a window is significantly linked to any other view that does not include buildings (p 0.003; IC 0.33–0.08).

#### 3.3.5. Modifications to the Home after Lockdown

**Figure 11:**
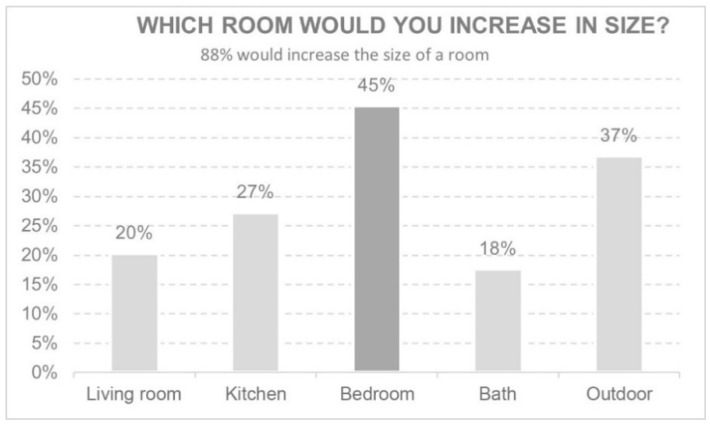
Which room would you increase in size?

This section considers the rooms which, after lockdown, those surveyed would like to increase the size of (Figure 11). In addition to modifying room size, 40% stated they would change the furniture or windows. The study also reveals that 98% of those who did not have a balcony or terrace would like to have had one.

## 4. Discussion

Flats were rated significantly lower than detached houses (both dwelling types were the most used during lockdown). However, while there were no differences between architecture and medicine students in the perception of imprisonment, this was significantly affected by the views of other buildings as opposed to any other view: park or sea or communal patio.

Among the students who felt imprisoned, a high percentage did not have a balcony or terrace but would like to have one. In contrast, those who felt peace did not express the need for a terrace or balcony.

It should therefore be highlighted that the environment (habitat) was decisive in the perception of imprisonment, as a feeling of peace was significantly more frequent among students who had views other than buildings (park, sea, etc.).

By no means of less importance in the appearance of neuro-psychiatric symptoms is the influence of the environment, or the setting of the home. A view of walls or nearby buildings has a negative effect on health, while the existence of open spaces such as parks and gardens protects against neuro-psychiatric symptoms. This finding could be of great interest to understand aspects such as anxiety or depression described in a similar population in a previous study. In addition to professional help for these pathologies, consideration should be given to the possibility of reducing their incidence through the better construction of homes and their environment [32].

Alcohol consumption during lockdown in this age group (59%) is similar to that reported in the national survey conducted by the Spanish Drugs and Addictions Observatory (OEDA) in the general Spanish population during lockdown (57%). It also confirms the decrease in pre-confinement consumption, which was estimated at 72% in this age group [60].

Weight gain has been reported in several studies during confinement [61,62,63] associated with an increase in the frequency and quantity of unhealthy foods. However, it is interesting to note that, in this young population, practicing any type of sport was not the main leisure activity, but rather the use of any device with a screen: tablet, mobile phone, computer or TV. The majority engaged in between one and six hours of physical activity per week. This attitude may have favoured the recorded weight gain associated with less physical activity.

In terms of excess weight and obesity, the health results reflect the values obtained in epidemiological studies in Spain. Obesity and excess weight have become the great epidemic of the 21st century, mainly affecting western countries. Perhaps in this sense, it is necessary to look at how architecture can help reduce the risk of obesity and if this is not possible, at least not increase levels. To do this, construction and residential developments should include open spaces within proximity, and both sport facilities and public gardens where aerobic exercise is not only limited to the communal patio. Access should be encouraged with comfortably designed, well-lit, decorated steps.

Regarding the specific characteristics of the home, the most desired was an increase in the size of the bedroom, influenced perhaps by the fact that being students, the majority of the time was spent in that room, especially in the case of shared student flats. The desire for a terrace or balcony to make use of during the confinement is also worth noting.

Obviously, this study had some limitations; first, the reduced number of participants prevented a more statistically robust analysis of the results. Secondly, it was carried out in one of the southernmost university cities in Europe, with a strong Latin character where life is usually lived on the street, which limits the extrapolation of results to other more central European cities. However, the joint analysis of personal and home aspects provides interesting insights into the influence of home design and environment on occupants.

## 5. Conclusions

The data obtained in the survey have led to various conclusions and reflections which offer answers to the objective set out at the beginning of this publication.

The first of these is that the students who participated in the study had a generally good evaluation of their homes; nevertheless, a high percentage showed neuro-psychiatric symptoms, which indicates that, although they were “comfortable” in the home, their health was affected negatively. Thus, it is possible that the home was not the only cause of these pathologies.

In line with the World Health Organisation’s [64] biopsychosocial model, the data obtained highlight the importance of the social aspect and how a lack of such leads to a decline in other factors, including the wellbeing of the individual.

The health issues suffered by people in confinement could have more to do with the loss of freedom rather than the dwelling itself. This argument gains plausibility when considering that this study was carried out among a cohort of young people of Latin character, used to personal contact, community and outdoor living.

As a consequence, it should be possible in the near future to design buildings with the necessary flexibility to respond to these and other needs of the inhabitants, either through the use of moving partitions, or the shared use of communal spaces. In fact, this should be one of the objectives that architecture addresses irrevocably, independent of the appearance of a new pandemic—or other similar global events—which forces the individual to spend long periods of time confined in their “contemporary caves”.

However, it could also be concluded that the aforementioned arguments concerning the intensive and exhaustive use of homes should never have taken place. It seems absurd that such an extreme situation as a general lockdown of the population had to occur for us to undertake a critical analysis of the private living space, to determine the ideal ventilation, natural lighting, dimensions, etc, and to test its impact on human health. 

In this particular case—architecture and medicine students—the impact of the home confinement has perhaps been stronger than in any other populational group, on the one hand, because of the characteristics of youth and, on the other, because of the special vision that these fledging architects and doctors have of the aspects of health and habitability conditioned by the type of dwelling in which they spent their confinement, and which has highlighted weaknesses and needs in their design.

Finally, it should be added that this method of surveying the opinion of “dwelling users” should be extrapolated to any other housing type. This does not usually occur, and a clear opportunity to advance the links between medicine and architecture is lost. If inhabitants’ comfort and health conditions were considered in future designs, they would have a positive effect on the quality of life of future generations. As the authors of this article concluded in a previous study [37], in these times of global pandemic and confinement, this is even more essential.

## Figures and Tables

**Table 1:** Characteristics of participants.

Participants		Age (Average and Standard Deviation)
Medicine	133 (70%)	24.64 (2.27)
Architecture	55 (30%)	24.35 (2.33)
Female	128 (68%)	24.65 (2.56)
Male	60 (32%)	24.35 (1.53)
Total	188 (100%)	24.55 (2.29)

**Table 2:** Results in health.

Results in health	n = 188 (%)
**Type of pathology**	
Digestive	60 (32.3)
Respiratory	18 (9.6)
Neuro-psychiatric	168 (89.4)
Infectious	42 (22.3)
**Neuro-psychiatric pathology**	
Migraine or Headache	70 (37.3)
Insomnia	93 (49.5)
Depression symptoms	92 (48.9)
Anxiety	106 (56.4)
Lack of rest	89 (47.3)
Irritability or bad moods	81 (43.1)
**Use of toxic substances**	
Hashish	2 (1.1)
Marijuana	7 (3.7)
Tobacco	28 (14.9)
Alcohol	110 (58.5)

**Table 3:** Results in comfort.

Results in comfort	n = 188 (%)
**Night-time noise**	
Nothing (0–1)	81 (43)
Low (2–4)	76 (40)
Moderate (5–6)	19 (10)
Substantial (7–8)	12 (6)
**Daytime noise**	
Nothing (0–1)	16 (9)
Low (2–4)	56 (30)
Moderate (5–6)	58 (31)
Substantial (7–8)	58 (31)
**Hours of natural light**	
Five or more	139 (74)
Four	17 (9)
Three	12 (6)
Two	7 (4)
One	6 (3)
No natural Light	7 (4)
**Need for artificial lighting**	
At the start and end of the day	120 (64)
All day	68 (36)

## Data Availability

Not Applicable.
